# Principles for Adapting Assessments of Executive Function across Cultural Contexts

**DOI:** 10.3390/brainsci14040318

**Published:** 2024-03-27

**Authors:** Matthew C. H. Jukes, Ishita Ahmed, Sara Baker, Catherine E. Draper, Steven J. Howard, Dana Charles McCoy, Jelena Obradović, Sharon Wolf

**Affiliations:** 1 International Education Division, RTI International, London E2 9FR, UK; 2 Graduate School of Education, Stanford University, Stanford, CA 94305, USA; iahmed2@stanford.edu (I.A.); jelena.obradovic@stanford.edu (J.O.); 3 Faculty of Education, University of Cambridge, Cambridge CB2 8PQ, UK; stb32@cam.ac.uk; 4 SAMRC Developmental Pathways for Health Research Unit, University of Witwatersrand, Johannesburg 2017, South Africa; catherine.draper@wits.ac.za; 5 School of Education, University of Wollongong, Wollongong, NSW 2522, Australia; stevenh@uow.edu.au; 6 Graduate School of Education, Harvard University, Cambridge, MA 02138, USA; dana_mccoy@gse.harvard.edu; 7 Graduate School of Education, University of Pennsylvania, Philadelphia, PA 19104, USA; wolfs@upenn.edu

**Keywords:** executive functions, assessment, culture, schooling, naturalistic settings, real-life measurements

## Abstract

Direct assessments of executive functions (EFs) are increasingly used in research and clinical settings, with a central assumption that they assess “universal” underlying skills. Their use is spreading globally, raising questions about the cultural appropriateness of assessments devised in Western industrialized countries. We selectively reviewed multidisciplinary evidence and theory to identify sets of cultural preferences that may be at odds with the implicit assumptions of EF assessments. These preferences relate to motivation and compliance; cultural expectations for interpersonal engagement; contextualized vs. academic thinking; cultural notions of speed and time; the willingness to be silly, be incorrect, or do the opposite; and subject-matter familiarity. In each case, we discuss how the cultural preference may be incompatible with the assumptions of assessments, and how future research and practice can address the issue. Many of the cultural preferences discussed differ between interdependent and independent cultures and between schooled and unschooled populations. Adapting testing protocols to these cultural preferences in different contexts will be important for expanding our scientific understanding of EF from the narrow slice of the human population that has participated in the research to date.

## 1. Introduction

Executive functions (EFs) are a set of higher-order cognitive skills that support self-regulation and goal-directed behavior [1]. They help children sustain attention by resisting distractions (inhibitory control–interference suppression), suppressing an immediate response to do what is more appropriate or needed (inhibitory control–response inhibition), holding and manipulating information in mind (working memory), and flexibly shifting among competing rules or demands (cognitive flexibility) [2]. EF skills have been linked to everyday behaviors that help children focus, plan, problem solve, and pursue goals. Ultimately, they are critical for the acquisition of knowledge and the formation of positive relationships [1], lay the foundation for later school success [3], and underpin resilience in high-risk settings [4,5]. EF assessments are helpful for appraising domain-general learning skills that underpin knowledge acquisition, rather than assessing the knowledge already acquired using domain-specific academic tests. Numerous interventions have been designed to improve EF and academic achievement [6].

This article focuses on direct assessment of EF skills—that is, measures of performance skills administered directly to children by an assessor. Compared with other methods of assessment, such as self- or adult-reported measures, direct assessments have the potential to be quicker to complete, to be seen as more objective and less subject to bias [7], and—through careful design—can more precisely assess specific EF skills.

In order for the results of direct EF assessments to be useful for understanding knowledge acquisition in everyday life, the tests should have ecological validity, defined as the functional and predictive relation between performance on a set of EF tests and behavior in a variety of everyday world settings [8]. The measures should capture how children deploy EF skills in everyday contexts in which they must face distractions, manage emotions, and receive support from others [9]. It is potentially more challenging to ensure ecological validity of direct assessments, compared with adult reports or observations, which are typically based on children’s everyday observable behaviors. Direct assessments, by contrast, involve gamified tasks that typically do not resemble children’s daily activities. Direct assessments of EF can show weak correlations with self-rated skills [7,10], arguably because the two assessment methods measure different mental constructs [11]. There is also an inconsistent relationship between direct assessments of EF and outcomes in everyday life [7]. Yet, other evidence finds that task-based measures of self-regulation are better predictors of current and future outcomes than parent reports [12].

The potential threats to the ecological validity of EF tasks have perhaps been overlooked. Although the interplay between universal, domain-general cognitive processes and cultural variations in cognition has been widely recognized [13], the assessment of EF has remained, until recently, influenced by its historical origins in neuropsychology [14]. That is, assessments of EF largely assume that these are universal, domain-general, individual brain functions and, therefore, that they do not vary across cultures. In this paper, we explore cultural preferences that may interact in important ways with direct EF assessments and that call into question the underlying assumptions of those assessment tasks.

One contextual influence on the ecological validity of EF assessments relates to the degree of shared cultural assumptions between those designing and administering the assessments and the children who participate in the assessments [15]. In other words, assessments of EF embody a set of assumptions held by those who created the tasks. These implicit assumptions can remain unspoken if they are also shared by the people involved in their use. If, however, the assessments are adapted beyond the contexts in which they were devised—for the most part, the rich, industrialized, educated countries of North America, Europe, and Australasia—there is a need to make the assumptions explicit in order to understand whether they are shared by participants in these new contexts.

The degree of this threat to ecological validity may depend on the everyday behavior the EF assessment is intended to predict. For example, a measure of EF skills that relies on children’s performance on standardized assessments will have a greater ecological validity when used to predict children’s performance on standardized academic tests than on children’s ability to complete everyday chores and activities. When ecological validity is potentially problematic, adaptations to assessment protocols should be considered.

The need for a basic level of adaptation of cognitive assessments is widely recognized [16]. Many developmental researchers are familiar with the need to pilot tasks to check for floor and ceiling effects and are skilled at adapting tasks to make them easier or harder for children in a given sample. Good psychometric test adaptation involves minimizing extraneous factors that may impede children’s responses. For example, if children do not understand the language being used to describe the rules of the task, then they will not be able to engage with the task. It is commonplace to adjust the wording of questions and instructions to ensure they are understood. Similarly, a basic step in task adaptation is to ensure that children are familiar with the stimuli being used. Many EF tasks (see Table 1) use animals and other familiar objects as stimuli—for example, the butterflies and frogs. It is standard practice to determine whether children are familiar with these animals before beginning the task. Overall understanding of language and stimuli in a task can be probed during piloting and with control questions at the start of a task that establish the child’s understanding. While these adaptations are essential, we argue that relevant cultural adaptations are likely to be needed beyond translating, adapting wording, and ensuring that culturally relevant examples are used [17,18].

There is a history of research that has highlighted the fundamental challenges—beyond the superficial issues of translation—in cross-cultural adaptation of cognitive assessment. Greenfield et al. [15] discussed three categories of challenges. First, the examiner and examinee may attribute different meanings to instructions and stimuli, even when the translation is good. Different values may result in contrasting opinions about what constitutes a correct answer. Second, some cultures view knowledge as being acquired by individuals, whereas others see knowing as a collective endeavor. Third, there may be different conventions of communication. For example, it may be more or less common for someone to ask a question to which they already know the answer or for a stranger to ask someone a direct question.

Ardila [20] examined, in detail, the values inherent in psychometric cognitive assessment. These values include the assumption that individuals are familiar with interacting one-on-one with a stranger; that the child recognizes the authority of the examiner; that the child is motivated to give the best possible performance; that the child accepts the isolated and artificial nature of the testing procedure; that the communication between examiner and examinee will be formal; that there is a shared understanding of the need to complete tasks at speed within a specific time period; and that specific testing elements—such as two-dimensional representations—are familiar to the examinee. Many of these assumptions of cognitive testing are shared by EF assessments.

The extent to which these assumptions are problematic for the validity of EF assessments is poorly understood because of the profound lack of evidence in developmental science outside of rich, industrialized countries [17,21,22]. There is good reason to question the assumption that the findings of research in WEIRD (Western, Educated, Industrialized, Rich, and Democratic) countries apply universally. Henrich et al. [23] review evidence that individuals in WEIRD countries are outliers on the spectrum of performance in several social, emotional, and cognitive domains of psychology. Evidence cited in this review highlights contrasts in performance between industrialized and small-scale societies and between Western and non-Western nations. Lancy [24] drew on anthropological evidence from 90 countries to argue that Western middle-class childhoods across the world have many similarities, including separation from the adult world, plus a focus on the individual and their cognitive competencies. This type of childhood contrasts with two other types—that of small-scale subsistence agricultural communities, where social responsibility to the social group is emphasized and children are integrated into the productive life of the community from a young age, and the childhoods of hunter-gatherer communities, which involve a high degree of freedom and autonomy.

The cultural contrasts described by Henrich et al. [23] and Lancy [24] guide our analysis of the ecological validity of EF assessments. We also recognize that cultural differences between societies have several additional layers of complexity. Preferences and assumptions differ within cultures, not just between them. Cultures evolve. In most contexts, values associated with urban, educated populations increasingly mix with those of subsistence agricultural communities as urbanization rapidly increases in many countries. It is likely that assumptions inherent in assessment procedures are shared by some participants in a community but not others.

Similarly, assumptions in EF assessments may be more or less shared with participants at different developmental stages. Some assumptions may be valid only with older children who have more experience outside one’s home or exposure to formal educational programs (e.g., pre-primary or primary school). There may also be an interaction between cultural and developmental stages—with assumptions becoming valid at different stages of development across cultures. In the following section, we aim to outline key assumptions of EF assessments that may not be shared by participants across cultures. Given the lack of research on the ecological validity of EF assessments across cultures, we draw on a range of evidence from developmental science, cultural psychology, and anthropology to identify what the potentially problematic assumptions of EF assessment paradigms are and how they may be addressed.

## 2. Cultural Preferences

### 2.1. Motivation and Compliance

Accurate measurement of EF, regardless of the paradigm or parameters, depends on participants’ willingness to adhere to and engage fully with task requirements. Thus, children’s performance on an EF task captures both their *skill* or competence/capacity in completing the task trials as well as their *will* or motivation to comply with the task’s demands. At lower levels of compliance, children’s performance on an EF task is less an estimate of what they *can* do (i.e., endogenous EF capacity) than a reflection of what they *did* do (e.g., score 0 on an EF task). A typical assumption of EF assessments is that the child is sufficiently motivated to invest effort in successful task performance, unless there is some overt indication otherwise (e.g., a child disengages). Yet research shows that, in some situations, children’s unwillingness to comply with EF task requirements and priorities can generate invalid estimates or no estimate at all (e.g., if the child entirely declines to respond)—sometimes in ways that can easily go undetected.

Willingness or motivation to engage with an EF task is influenced by intrinsic factors, such as whether the participant finds a task enjoyable or feels personal satisfaction in performance, as well as extrinsic factors, such as social praise, approval, or rewards. Research shows that intrinsic and extrinsic motivation are not mutually exclusive constructs and that children are motivated both by their interest and by incentives [25]. EF assessments are often designed to leverage both types of motivation to increase children’s engagement and compliance with task instructions. Assessments may look and feel like fun games. They may be administered with social praise and encouragement during the learning and practice phases and with small gifts, like stickers, upon task completion. In general, these task incentives are assumed to be effective in motivating children to comply with assessment requests—at least in WEIRD contexts—although their impact on performance has not been systematically studied.

There is evidence that this assumption—that the design characteristics of EF tasks are sufficient to ensure a child’s compliance with its requirements—may be problematic beyond (and also within) WEIRD contexts. Research using delay-of-gratification tasks, in which children are asked to wait for some enticing outcome or reward (sometimes involving a choice against a smaller but immediate reward), has revealed that children’s perception of the assessor’s trustworthiness can affect how long they wait. Participants who are promised a second, delayed treat by a trustworthy adult wait significantly longer than those tested by an unreliable adult [26,27,28]. Looking cross-culturally, children’s EF task performance has been shown to vary with their perception of group membership and group norms. Participants do better if they are told they belong to a group that performs well on the task [29,30].

Continuing with the example of delay-of-gratification tasks—which can be seen as a test of children’s compliance with adult instructions—cultural preferences may influence performance on this task several ways. Lamm et al. [31] found that children of Nso farmers in Cameroon were better able than German children to wait for a second treat in a delayed-gratification task. They also exhibited few of the signs of an internal battle of wills—for example, sitting on their hands or looking away from the treat—that were seen among their German counterparts. The authors argued that the Nso children were comfortable being compliant with an adult’s directives as a result of their sense of belonging and perceived responsibility to the group. The implication is that Nso children are compliant with adults across domains. By contrast, Munakata et al. [30] highlighted the role of domain-specific experience and its effect on compliance. They found that children in the United States were able to effectively delay the gratification of opening a gift, because they were practiced in doing so. Japanese children performed less well at this specific gift-delay situation but were comparatively better able to delay the gratification of eating food because waiting for a whole group—such as a class of children—to be ready before eating begins is a common practice in Japan. Thus, the tasks need to be adapted to take into account cultural expectations for behavioral self-regulation (e.g., waiting). While children’s performance on delay of gratification tasks have been used as a measure of inhibitory control, only tasks that are designed to elicit effortful delay of culturally relevant impulse should be considered valid measures of EF. Further, researchers should interrogate how issues discussed above may affect children’s performance on other EF paradigms.

There are other examples of how engagement with EF tasks is affected by cultural norms around appropriate behavior. For example, in rural Pakistan, preschool children refused to obey task instructions issued by a puppet, because they found this form of presenting the requests unfamiliar. Instead, the children remained motionless, which yielded invalid performance scores (see Appendix in Obradović et al. [32]). Yet, young children growing up in Western contexts are often socialized by their families and by media programs to see talking puppets as approachable and fun, and so puppets have been used in the design of some EF tasks to increase motivation, comprehension, and compliance. Further, in cultures that value learning by observing and pitching in [33], such as the Yucatec Mayan culture, children may not feel it is appropriate to attempt activities that are beyond their ability or that they have not first observed adults do [34,35]. In contrast, U.S. children are often socialized to see the value in attempting activities beyond their skill level, with adults’ scaffolding and encouragement [34].

Further, in cultures where children are expected to comply with adult requests as a sign of respect for authority, higher levels of compliance with an assessor’s requests may not translate into accurate assessment of endogenous EF capacity. For example, children may comply by making a forced selection (e.g., pressing a button on a tablet task) even when they do not understand the task rules and do not feel comfortable asking clarifying questions. This disconnect highlights the need to interrogate the validity of data, especially if the response pattern seems perseverative and does not vary in response to the task’s demands and instructions.

On the other hand, compliance tendencies can be harnessed in ways that promote EF skills. Among young children from Central Europe and Melanesia, Rybanska et al. [36] found that ritualized versions of school activities that promoted EFs using firm rules and established expectations (e.g., “It has always been done this way”) were more effective than instrumental versions of the same activities that included explanations (e.g., “If we do it this way, we will learn”). Further, experimental manipulation of the value of the assessed skills or the need to serve as an exemplar has been linked with higher performance in children from the United States [37,38,39,40].

Cultural expectations and values can thus be leveraged to optimize performance as long as they do not invalidate the key assumptions of EF paradigms. Specifically, measuring behaviors that have been socialized as dominant or prepotent responses in a given culture (e.g., sitting quietly, waiting patiently for food) is not a good way to assess EF skills, which, by definition, require cognitive effort. Instead, it may be more prudent to select EF tasks in which prepotency is built into the task (such as Go/No-Go paradigms, in the case of inhibition tasks) rather than assumed to be present in the local culture. It can also be beneficial to select for or adapt EF tasks and elements (e.g., stimuli) that ensure they are contextually acceptable and appealing (i.e., children want to engage in the task).

To further support these EF task selection and adaptation decisions, future studies need to examine how a child’s subjective perceptions of EF task demands, the effort required, the assessor’s demeanor, the assessment context, and the participant’s current mental or physical state (e.g., stress, hunger) affect their engagement and compliance with task procedures. For instance, it is important to understand how acute stressors and challenges, such as hunger, illness, tiredness, and stress, may further undermine children’s will and ability to apply their skills, especially because these health issues tend to co-occur with other inequities that undermine performance on standardized assessments [41,42,43]—factors that can vary systematically across contexts. In parallel, it is important to understand how factors that promote engagement in one culture (e.g., being awarded “points” for performance, which otherwise have no inherent value) may have little effect or may even undermine task engagement in another culture. Equipped with these insights, researchers can focus on designing or adapting EF tasks to use children’s cultural experiences and values to increase motivation and compliance in a way that maximizes the reliability, validity, and comparability of the assessed EF capacity.

### 2.2. Cultural Expectations for Interpersonal Engagement

Children’s development occurs within particular groups and bounded communities, the expectations of which vary across cultures and contexts. Many widely used EF assessments entail an adult assessor interacting one-on-one with a child, administering a battery of tasks or games directly or through a tablet. Such arrangements make important assumptions about children’s expectations regarding social situations, including that children are comfortable and can perform optimally when interacting with an adult stranger who is asking them to complete a series of novel, often abstract tasks. Yet, for some children, these sorts of social situations may be very unfamiliar, leaving them feeling uncomfortable and fostering unnatural conditions that lead to poor or otherwise nonrepresentative performance.

For example, children in urban middle-class families across multiple countries have been documented to participate in other adult-managed, child-centered activities (e.g., Rogoff et al. [44], Coppins and Rogoff [45]), making such interactions familiar and comfortable. In contrast, children in Indigenous rural communities such as the Yucatec Maya were found to rarely spend time alone or one-on-one with adults and instead tended to participate in more collaborative, family- and group-centered activities [35]. Similarly, research with Latino families in the United States has highlighted the strengths associated with viewing children’s learning and motivation as situated within communities that exercise cognitive demands and social expectations, advancing particular forms of cognitive growth that are embedded within social participation and the motivated desire to become a competent member of a broader social group [46]. Indeed, groups provide opportunities for transfer of learning to individuals through cognitive and social processes that arise during interaction, and individual members share and combine knowledge through feedback, help, experimentation, and simultaneous work [47].

Even when children are familiar with the situation of working one-on-one with an adult to complete a task, their expectations regarding the content and structure of these dyadic interactions may vary across settings. For example, research has shown culture-based variability in children’s reactions to strangers, with children from Germany showing more initiative during interactions with unfamiliar adults than children from Cameroon [48]. Similarly, research using book-reading tasks has shown that in cultures where parents use more restrictive and discipline-oriented interaction styles, conversations between parents and children tended to focus on more concrete subject matter directly related to the book being read, whereas in contexts where parents use more responsive styles, conversations reflected more abstract content related to the child’s experiences [49].

Existing direct- and tablet-based assessments administered one-on-one by adults are assumed to be “neutral” ways of measuring children’s underlying abilities, because they are expected to eliminate distractions or other confounding influences that can arise under less structured situations. However, this assumption of “neutrality” is called into question if dyadic assessments are premised on unnatural and unfamiliar social dynamics that are vastly different from the social experiences children face in their daily lives. An adult (stranger)–child dyad, where adults direct the task for children to complete, makes assumptions about children’s comfort and experience with engaging in such a dynamic.

Such a setup is likely to favor White, Western, middle-class children who are used to engaging in adult-assigned tasks on their own, in a more contractual arrangement (e.g., completing a chore to receive an allowance) [45]. In rural communities, as well as in families with a collective cultural heritage who live in urban areas, children seldom spend time by themselves or in dyads and are almost always in the company of adults and other children [34,45]. As such, asking a child to complete an EF task in the presence of a single adult will likely work well for some groups of children and not others. An example was described by Alcalá and Cervera [50], who tried conducting semi-structured interviews with children in a small rural community in Yucatán. One-on-one interviews yielded very little from the children, because they were uncomfortable sitting alone with an adult across a table, looking an adult in the eye, and answering their questions. Two solutions yielded richer data from children. The first was having small-group interviews, with children helping each other elaborate on their responses. The second solution emerged after the researchers had spent over a year in the community. After the children became more accustomed to the researchers, one-on-one interviews became more feasible, but nevertheless required sitting side-by-side and avoiding direct eye contact [50].

Future research could assess children’s skills on an EF task both one-on-one and in a group setting where other children either do or do not provide input. Using these parallel approaches would yield complementary insights into children’s EF skills under different social and cognitive demands. In particular, tasks administered in group settings could generate information about children’s EF skills that is more ecologically valid and reflective of the sorts of skills needed for success in daily life within one’s community. Research from the US supports this hypothesis. Indeed, one study comparing the same children’s performance on the same assessment administered one-on-one versus in the presence of peers found that only the group-administered assessment predicted improvements in children’s academic performance [51]. This study suggested that group-based EF tasks may capture unique information about children’s regulatory capacities in the everyday environments where broader learning and development are fostered.

Beyond improving ecological validity, group-based EF assessments could also yield novel insights regarding the extent to which regulatory processes may operate at a collective, rather than exclusively individual, level. Developmental psychologists have long hypothesized that children develop self-regulatory capacity over time through “other regulation”, or scaffolded support from primary caregivers [52]. Emerging theories have also hypothesized the relevance of “collective regulation” in more collectivistic cultures [18]. Nevertheless, empirical research examining social dimensions of regulation and EF remains extremely limited. Future research focusing on novel EF paradigms intended to capture group-level or otherwise collaborative approaches to regulation could help to fill this knowledge gap.

### 2.3. Contextualized vs. Academic Thinking

Many EF assessments assume that performance on decontextualized tasks is similar to performance on everyday tasks. For example, digit-span tasks assume that being able to remember an abstract sequence of digits is a good proxy for verbal working memory in everyday life. A decontextualized task may differ from an everyday task along at least two dimensions, however. It may use abstract representations instead of real objects, and it may lack structure and meaning that would be inherent to the task in everyday life. For instance, it is unclear the extent to which recounting a random string of digits recited by a tester corresponds to everyday uses of working memory, such as recalling the multistep instructions a parent gives to a child. In such cases, children may be identified as having a poor working memory, when, in fact, they have a good working memory for everyday things with which they are familiar.

Research in cultural psychology suggests that cognition is adapted to context. Children become skilled at manipulating abstract concepts and tackling decontextualized tasks if they have experience of doing so, rather than because these tasks draw on a fundamental domain-general ability. Evidence suggests that this experience is largely acquired through schooling. Indeed, Greenfield et al. [15] suggested that the degree of formal schooling may be *the* largest factor determining whether a culture shares the assumptions and conventions of most ability-assessment paradigms.

Several lines of evidence suggest that schooled populations are more familiar with some types of decontextualized cognition. Unschooled individuals fare less well when trying to recall unconnected information because they are less likely than schooled individuals to spontaneously employ strategies to aid recall, such as rehearsing, elaborating, or categorizing items [53,54,55]. In Indigenous hunter-gatherer communities, those with experience of schooling employed working-memory strategies associated with rote learning—such as primacy, recency, and serial clustering—whereas individuals with no schooling organized recall in semantically meaningful categories [56]. Similarly, several studies have suggested that unschooled populations show good recall of items when they are organized by meaningful schema. For example, compared to children from the U.S., Guatemalan Mayan children performed slightly better in reconstructing the position of 20 objects placed around a model village [57] but performed less well in recalling a list of isolated items [58]. The interpretation was that children with formal schooling have more practice than unschooled children in imposing meaningful schema on lists of isolated items. When a meaningful schema is provided—such as the locations in a model village—unschooled children show strong recall.

When presented with syllogisms, unschooled populations prefer to reason based on experience rather than restricting themselves to the information provided in the task [59]. Lave [60] found that Liberian tailors’ experience with formal schooling was related to their ability to solve abstract mathematical problems of the kinds posed in school, and tailoring experience was related to the ability to solve practical mathematical problems of the kind found in tailoring.

Children may also have different levels of familiarity with abstract representation of objects. Children in Zambia performed better on a nonverbal reasoning task when patterns were presented using three-dimensional objects rather than two-dimensional pictures [61]. Performance on the two-dimensional task was significantly associated with children’s, and the children’s parents’, experience of formal schooling. Similarly, children in Scotland outperformed Zambian children in a task requiring drawing an object but Zambian children outperformed Scottish children when required to reproduce an object using wire [62].

The Zambia/Scotland study [62] illustrates the general principle that performance on assessments is enhanced when children are familiar with the objects, concepts, and strategies involved. Formal schooling provides a specific example of this principle. That is, schooling involves a set of relatively similar experiences for children globally—including exposure to two-dimensional representations and the manipulation of information divorced from its context.

When researchers review the design of EF tasks to consider how to address the challenges of assessing children with little experience of schooling, they should consider the goal of the assessment. If the assessment aims to measure the deployment of EF in ways conducive to academic readiness and performance, it is appropriate to include abstract, school-like tasks. In fact, the abstract nature of the task may be integral to the competency being assessed. Making a test more contextualized may reduce difficulty levels as well as the test’s ability to predict school performance.

If, however, the aim is to measure the application of EF to everyday functioning more generally, including in common situations that occur in daily life, the use of abstract school-like tasks may be less valid. In such cases, validity could be improved by the use of real-world scenarios and/or stimuli in EF tasks with which children are familiar. Such adaptations could involve relatively modest changes to test procedures. For example, an adaptation of the Corsi Block test—a spatial working-memory test that involves remembering the order in which blocks are tapped by an assessor—could use objects from the child’s environment rather than blocks. Verbal working memory could be assessed through an everyday task such as remembering a shopping list.

The above examples involve introducing real-life stimuli into an EF task, while retaining the format of an adult assessing a child in a testing procedure conducted in a separate dedicated space removed from everyday life. Other assessments aim to replicate real-world scenarios with naturalistic tasks [63]. One example is the Multiple Errands Test [64], which was designed to assess the impact of EF deficits on everyday function. In the test, participants are given a list of several actual errands to complete, requiring them to plan, sequence, prioritize, and adapt to unexpected situations. Their performance is assessed using an observation protocol. Examples of similar assessments developed for children include the Children’s Cooking Task [65], in which children are assessed for the number of errors they make in a cooking task involving setting and maintaining goals and multitasking, and the Do-Eat Assessment Tool [66], designed to measure a broader range of cognitive and motor skills by observing children conducting a sequence of activities, including preparing food.

Future research could compare individuals and populations with different histories of formal schooling, presenting them with contextualized (concrete) and decontextualized (abstract) working-memory tasks to assess whether they have different loadings on a latent working-memory factor. More generally, assessments could be adapted to unschooled populations so that decontextualized tasks with abstract stimuli are replaced with meaningful everyday tasks. In such adaptations, it is important to bear in mind that EF tasks are designed to assess effortful processes that may rely on the novelty of a task. Thus, making a task more familiar may reduce a task’s cognitive load (e.g., a list of familiar objects can be “chunked” into a shorter list of categories) or its potential to capture effortful processes [67,68]. Research could aim to assess the degree of effort—for example, through self-report—when respondents are carrying out contextualized, everyday EF tasks versus tasks in more artificial testing scenarios.

### 2.4. Cultural Notions of Speed and Time

Implied in many cognitive assessments is that the speed with which a task trial is completed (assuming that it is also done with accuracy) indicates higher cognitive ability. Recent work illustrates how the speed–accuracy trade-off varies across developmental stages and types of cognitive tasks and that speed has inconsistent relation to accuracy even in Western samples [69]. Debates around this association have tried to explore the extent to which speed of cognitive processing is a unitary construct and is universal [70,71]. Although response-time measures (as an indicator of intelligence) may have typically been seen as free of cultural bias, recent research has recognized that different cultural notions of mental speed can influence performance on these tasks, and that context needs to be carefully considered [72]. There has been increasing acknowledgment of the complexity of the relationship between speed and intelligence (to which EF is an important contributor), and that there are cross-cultural differences [73,74] and similarities [71] in this relationship.

Cultural notions of time will likely have an influence on how speed (response time) is understood in relation to cognitive assessments. These cultural notions include time orientation (future, present, or past orientation) [75,76] and pace of life. Economically productive and individualistic countries often expect and have a normative faster pace compared to economically undeveloped and collectivist countries [77].

Approaches to time across different cultural contexts are also relevant, and “clock” and “event” time have been identified as two contrasting approaches, i.e., measuring time by clocks, versus measuring time by social events. Other differing approaches are monochronic time and polychronic time. A monochronic approach is associated with a preference for doing one thing at a time and is characterized as more adaptable. Here, time is linked to social interactions, an approach that is common in event-related cultures. A polychronic approach involves a preference for doing several things at once, with time seen as more fixed and linear. This approach is more common in cultures that measure time by clocks [75].

Different approaches to time could be related to cultural notions of mental speed, as suggested by research on associations between response time and intelligence in industrialized versus non-industrialized countries. In “by-the-clock” (industrialized) cultures with a more linear approach to time, “mental speed may be a valued characteristic that is highly internalized” [72] (p. 215), whereas in event-related (non-industrialized) cultures, mental speed may not be strongly linked to intelligence, and may be a less valued characteristic [72]. However, cultures may demonstrate within-community differences, as shown in a Ugandan study that found that community members associated intelligence with being slow, careful, and active, but teachers in the same community associated intelligence with speed [78].

In relation to EF assessments, designers often assume that children who can do timed tasks faster and under pressure have better EF and those who are slower have poorer EF, given similar levels of accuracy in performance. This assumption may be embedded in some untimed EF tasks as well—for example, decay of trace in some working-memory tasks—such that by design and scoring, those who are slower will perform worse (and thus have poorer EF). Furthermore, there may be an assumption that the notion of time (and hence speed) is perceived similarly across cultures, resting on the assumption that the speed of doing things is valued similarly across cultures. However, based on the cultural considerations discussed above, this assumption should be challenged. In addition, these considerations raise the question of whether in certain cultural contexts, it is better to do things quickly or to do things correctly, even if correctness takes longer.

On timed tasks, among children from event-related cultures and/or with cultures that opt for a monochronic approach to time, speed may be valued less highly, and other cultural priorities may take preference, such as accuracy or social interactions. On these tasks, children might perform more poorly because they are more inclined to take longer and be sure they have what is perceived as the correct response, depending on their cultural context. In certain cultural contexts, the consequences of getting something wrong may be unpleasant and serious (e.g., harsh punishment of children, disrespect from community members, stigmatization), which could influence children to prioritize accuracy over speed in an assessment situation and could influence their motivation and compliance when they are completing tasks, as discussed in Section 2.1. Therefore, in a trade-off between speed and accuracy, speed may tend to be deprioritized.

The evidence presented in this section does not suggest that response time in EF assessments should always be disregarded; there may be instances and contexts where timed tasks are appropriate. However, it would be important in cross-cultural contexts not to rely on response time as a sole indicator of cognitive ability, or to overestimate its relationship to intelligence. EF assessments should therefore carefully consider this assumption before relying heavily on faster response times as indicators of better skills. In support of this point, a study on adult EF across diverse global contexts found that reaction time plus accuracy provided a more reliable measure of EF, compared to reaction time alone [74]. Future research could investigate the validity of reaction time and accuracy measures in populations with different notions of time (e.g., urban vs. rural communities in one country). Similarly, research could explore whether untimed tasks (such as some versions of the Stroop task) have more universal ecological validity than timed tasks designed to measure the same construct (such as the Go/No-Go task, also designed to measure inhibitory control—see Table 1). Researchers could also conduct qualitative research to explore these contrasting notions of time through ethnographic observations as well as in-depth interviews in order to better understand how time (and hence speed) may fare against other more salient priorities (e.g., doing a task well) in these different cultural settings.

### 2.5. Willingness to Be Silly, Be Incorrect, or Do the Opposite

Direct assessments of children’s EFs often require children to do or say something that is “silly” or incorrect in order to deviate from what is expected and draw on greater cognitive load. In order to measure inhibitory control, children are expected to do something that is the opposite of what they would typically do, to demonstrate their ability to suppress their dominant response. For example, in Stroop tasks, children need to be willing to say something that is incorrect (e.g., saying “day” when shown a picture illustrating night) or atypical (e.g., saying the ink color instead of what the printed word says). In Head-Toes-Knees-Shoulders, children need to be willing to do something that is incorrect (e.g., touch their knees when told to touch their head).

Tasks also require children to be willing to deviate from what the assessor does and *not* imitate an action. For example, children need to do something in the reverse order of what is shown to them for backwards Corsi block/dot matrix and digit-span tasks that measure working memory. For Peg Tapping tasks that measure inhibitory control, children need to do something different from what the assessor did (e.g., tap once when the assessor tapped twice).

In some cultures, children may not be willing to say something that is incorrect or the opposite of what it should be. Children with cultural unfamiliarity with doing something that is silly/incorrect or doing something that is the opposite of the assessor may score lower on EF tasks with this assumption, which would not reflect their EF skill. It is a cultural assumption of the task that does not align with their cultural norms.

An unwillingness to be silly/incorrect or do the opposite may be found in cultures with norms of following what is expected and imitating what is observed. As referenced in earlier sections on compliance and obedience, children may be expected to follow the norms of the collective society and not perform actions that deviate from what is expected. Typical child–adult interactions may also involve children imitating adults to learn from them [33]; hence, doing something the opposite of an adult may be an unfamiliar concept for them.

Although children universally engage in pretend play, which involves deviating from reality and what is expected, there are differences in the content of the play and who children engage with in pretend play [79,80,81]. Children across cultures base pretend play on their experiences, which vary across cultures [82]. Across six cultures based in subsistence communities, children’s pretend play was more focused on “work–play” activities where children imitated skills and activities in a fun way [83]. Therefore, children may be less familiar with EF task demands of pretend activities that go beyond imitating skills and require children to deviate from reality.

Further, outside of Western contexts, children are more likely to engage in play with peer groups than with adults. Parents and caregivers in many communities may be more involved in economic activities and not have time to engage in play with children [81]. Children not familiar with engaging in pretend play with adults may be less willing to engage in adult-directed tasks that require them to do something silly or playful.

There may also be a cultural norm of avoiding incorrect statements because that is associated with lying. Among the Yucatec Maya, children did not perform well on Stroop tasks because the tasks went against their cultural norms of avoiding untrue statements (e.g., calling the sun “night” [34,82]). Children who are more familiar with abstract concepts and school-based thinking may naturally perform better on these tasks because they are familiar with following instructions for the purposes of completing an arbitrary objective.

Based on initial piloting and cognitive interviews, if children are okay with doing something that requires them to be silly/incorrect or do something the opposite, then those tasks can be administered. If children are unwilling to follow those task assumptions, then other EF tasks can be used instead that do not require those actions. Examples include Go/No-Go for inhibitory control, Hearts and Flowers for inhibitory control/cognitive flexibility, and a Self-Ordered Pointing Task for working memory (see Table 1).

Future researchers can experiment with introducing children to tasks by asking them to imagine that they are in a different world and then setting up a pretend play scenario where they may be more willing to accept the assumptions of Stroop tasks and other assessments that require children to be silly or do something that deviates from reality. Additionally, these tasks can be administered in a group of children to create a play environment that may represent more typical peer-based play interactions that children are more familiar with. Creating a pretend play environment when administering tasks where children need to be silly/incorrect/opposite of what is expected may engage them in the task despite any deviations from cultural norms.

### 2.6. Subject-Matter Familiarity

One basic assumption of EF assessments is that the content or subject matter should not affect the validity of the task, as long as they are understood by participants. This assumption was not borne out in experiments aimed at testing the “thematic materials effect”. These experiments showed that participants’ logical inferences can differ because of their cultural experience of the subject matter [84]. For example, British participants with experience of specific postal rules were much more likely to follow sound logical reasoning (“if p, then q…”) when they were asked to enforce such rules than American participants who had no experience of those specific postal rules [84]. These findings suggest that knowledge and experience interact with domain-general executive processes in a bi-directional way. When rich knowledge is activated in a task, sparking meaningful links to relevant examples and counterexamples in one’s own experience, the executive demands may be lower, compared to when knowledge is thin in relation to the subject matter. In cases where there was no prior familiarity with the subject matter, there would be no automatic cueing of relevant information to support the reasoning, and with the additional effort in inferences, the executive demands might be higher.

## 3. Cultural Preferences and the Ecological Validity of Different EF Subtasks

Typical EF assessment batteries involve several tasks assessing a range of skills (Table 1). Inherent in each task is a set of assumptions that may align with, or be inconsistent with, the cultural preferences of children being assessed. In this section, we discuss in greater depth how each of the cultural preferences described above may interact with the assumptions of different EF tasks.

Some cultural preferences are likely to affect performance across a range of tasks. All tasks involve the assumption that children will be motivated to comply with the assessor’s instructions. Thus, cultural variations in compliance with an assessor may affect performance on all tasks. Similarly, a typical EF assessment protocol assumes that the child will be comfortable with a one-on-one interpersonal interaction. A cultural preference for collaborative engagement and group-based activities will thus affect performance on all tasks.

Other cultural preferences can affect some tasks more than others. Individuals from cultures with a monochronic perception of time, where speed is less valued, may perform poorly on tasks that use reaction time as a measure, such as tablet-based versions of the Hearts and Flowers or Silly Sounds Stroop Task. However, to the extent that all tasks in an EF assessment battery are time-bound, this cultural preference may influence performance on all tasks. Similarly, tasks may vary in the degree to which experience with schooling is assumed. In the discussion above, we identified two key dimensions of academic thinking. The first involved the ability to tackle tasks that were divorced from context. This ability may be beneficial across all tasks, but its effect has been most clearly demonstrated for working memory tasks [53,54,55,56]. The second aspect of academic thinking involves familiarity with abstract representations of stimuli. This aspect could affect performance on any task with two-dimensional pictures.

Finally, some cultural preferences have relevance only for specific tasks. Of the tasks listed in Table 1, willingness to be silly or do the opposite is relevant only to specific tasks, such as the Stroop paradigm and the Backwards Digit Span task. Another specific effect of a cultural preference discussed above is that children are better at resisting temptation for specific things, such as food or gifts, if the norm in their society is to delay gratification for them [19].

## 4. Principles for Organizing Cultural Preferences

Our analysis so far has treated cultural preferences separately. However, these preferences are not independent. Some organizing principles have been proposed [85,86] that help us to recognize the relationships among cultural preferences and to see culture as a system. The principles also help to understand where in the world—in which societies—we might expect to find the cultural preferences. Such predictions about where cultural preferences are found cannot take the place of primary research when EF assessments are adapted to a new population, but they can provide hypotheses to guide this research.

### 4.1. The Independent and Interdependent Selves

One set of organizing principles for cultural preferences was described succinctly by Markus and Kitayama [86] as the contrast between the independent and interdependent self. Keller [85] made a similar distinction between the constructs of psychological autonomy and hierarchical relatedness. According to Keller [85], Western middle-class childhoods are guided by the principle of psychological autonomy (similar to Markus and Kitayama’s [86] notion of the independent self). This principle results in children learning to act autonomously based on personal preferences, intentions, and other inner states. Individuals view themselves as self-sufficient, distinct, and unique [86]. This model prioritizes mental states that foster self-enhancement and personal expression. This cultural model emerges from WEIRD child-rearing strategies [85,87,88], where abundant resources and smaller families allow greater investment in children’s cognitive development. The nature of urban life, where encounters with strangers are common, fosters this emphasis on individuality. The principle of psychological autonomy is adaptive where economic productivity relies on educated individuals with unique skills.

The second principle within this set is that of hierarchical relatedness (similar to Markus and Kitayama’s [86] notion of the interdependent self), which is typical of small-scale subsistence agricultural communities. In this model, communal goals take precedence over individual autonomy. Society is organized hierarchically, with deference to older—and other senior—members of society. Individuals have autonomy but are driven to act in the interests of the collective by the sense of well-being [89] and belonging [46,90] that it gives. This cultural model arises from child-rearing strategies in subsistence farming communities [85,87,88]. In these communities, the pursuit of communal goals, typically within family units, is the key to economic productivity, and village life involves maintaining long-term relationships rather than many brief encounters with strangers.

The notions of independent and interdependent self (or psychological autonomy and hierarchical relatedness) map onto several of the cultural preferences discussed in our analysis. In interdependent cultures, expectations are that interpersonal engagement takes place in groups rather than dyads [45,91]. Such cultures also involve hierarchical relationships in which obedience to authority is emphasized [92,93]. Thus, compliance to assessor instructions is likely to be high. This focus on compliance also suggests that individuals in interdependent cultures may be less willing to be silly or say the opposite in a Stroop task. Also, in interdependent cultures, there is less emphasis on developing individual creativity and, as a result, parents are less likely to engage children in pretend play, which in turn may mean children are less accustomed to being silly with adults [34,82].

Arguably, the independent/interdependent distinction also has relevance for cultural preferences related to the perception of time. Broadly, interdependence is associated with a greater focus on people, whereas independence is associated with a focus on tasks, with an associated preoccupation with clocks as a means of telling the time [75]. If this pattern is accurate, it would imply an association between independent cultures and polychronic time, although factors such as climate/seasons and the prevalence of time-keeping technology are also likely to influence the cultural perception of time.

If the independence/interdependence models of the self underlie many of the cultural preferences we describe in this article, we can draw on extensive literature to understand where—in which societies—these preferences may be found. As discussed above, the interdependent construal of self is associated with small-scale communities that practice subsistence agriculture [24,85]. Because culture changes slowly, it is likely that similar values can be found in societies that only recently moved away from subsistence agriculture. However, over time, a cultural shift toward independence can be driven by sociodemographic factors, including urban life, commerce, education, and technology [88,94,95,96]. Keller [85] described a hybrid cultural model in which education, urban life, and employment in commerce can lead individuals from a hierarchical, related (interdependent) culture to develop greater psychological autonomy while still retaining a strong sense of relatedness to the social group.

The contrast between small-scale subsistence agricultural communities and more Western societies is well characterized but is only part of the explanation of the global distribution of independent and interdependent cultural models. Interdependence is common in East Asia—including in countries such as Japan with a long history of industrialization—as well as in other Asian, Latin-American, African, and south European cultures [86]. And there are other populations, besides the Western middle class, whose values contrast with those of agricultural communities. Research in Turkey [97] found greater social interdependence among farming and fishing communities than among herders. There are many variations, too, in the independent construal of self across Western countries (see Harkness et al. [98]).

Finally, we recognize that many other factors influence cultural preferences, such as religion and regional differences in values [99]. Cultures interact and influence one another increasingly with the forces of globalization [100]. However, we argue that the contrast between the cultures with independent and interdependent selves is a useful first-order proxy for understanding the origin and distribution of several cultural preferences.

### 4.2. Formal Schooling

An additional factor that shapes cultural preferences is experience with formal schooling. As discussed in Section 2.3, schooling is one factor driving the emergence of the values of independence [88,94]. In addition, familiarity with schooling may influence the cultural preference of academic thinking discussed above, including the capacity for contextualized thinking and the ability to understand abstract stimuli.

It is understandable that skills acquired through schooling may be mistaken for universal skills, given that schooling is near universal in the WEIRD contexts in which most psychological research takes place. However, formal schooling is not universal for most of the world’s population. Only 63% of school-age children in sub-Saharan Africa complete primary education. The rate is 55% in low-income countries globally [101]. Learning poverty—the proportion of 10-year-olds who cannot read a simple sentence—is estimated to be around 90% in sub-Saharan Africa and close to 80% in Latin America, the Caribbean, and South Asia, with a global average of 70% [102]. Worldwide, 13% of adults are not literate [101]. Of the 222 million children affected by global crises, 78 million are out of school [103].

When working with out-of-school populations, researchers and practitioners cannot assume children are familiar with the decontextualized academic thinking, or the strategies and stimuli of the classroom. If these vulnerable children are to benefit from research and interventions related to EF, assessments need to be creative in finding ways to improve ecological validity of tasks for unschooled children.

### 4.3. Implications for Research and Practice

Many of the cultural preferences identified in this paper are underpinned by two principles. The first principle involves a contrast between cultures that emphasize an independent self versus those that emphasize the interdependent self. The second involves a contrast between schooled and unschooled populations. It is critical to note that most EF research and practice to date has taken place in cultures that emphasize independence and where formal schooling is widespread. Very little research has taken place in cultures that emphasize interdependence or in unschooled populations. This gap implies that existing research and assessments may have limited applicability in these populations, which constitute most of the world’s population. The assumption that EF research is universally applicable is not plausible and underpins the need to bolster EF research in the Majority World [104].

It is also important to note that these two key principles—related to the construal of self and to schooling—do not capture all of the cultural preferences discussed in this paper. The adaptation of EF assessments likely involves consideration of many other locally specific factors. Several examples of such adaptations are discussed above. One such example is the cultural variation in types of treats for which children are condition to wait—for food in Japan and gifts in the United States [29,30]. Another example is that children respond well to instructions from puppets in many countries but did not in a study in Pakistan [32]. Similarly, children’s familiarity with specific subject matter and stimuli is likely to vary context by context.

## 5. Discussion

The main thesis of this article is that EF assessments developed in one context embody several assumptions that may not hold when researchers are working in a different context; thus, the assumptions may threaten the ecological validity of the assessments. While many researchers take steps to address the most salient issues of adaptation—such as ensuring that children understand instructions and recognize the stimuli presented to them—many assumptions of assessment protocols are implicit and therefore not routinely addressed. In some cases, these assumptions can have fundamental implications for the meaning that children attribute to the tasks.

We identified six cultural preferences, organized in large part (but not exclusively) by two higher-order principles, which may be incompatible with the assumptions of EF assessments. Children from cultures with an interdependent—as opposed to independent—construal of the self may be more compliant with assessor instructions; may prefer group-based assessments rather than those based on one-on-one interactions; and may be uncomfortable being silly or saying the opposite in front of an adult. Children with less experience of formal school, or from communities where schooling is not the norm, may have difficulty with tasks that are divested from their context and have abstract representations of stimuli. It is striking to note that almost all research into EF so far has been conducted in societies that emphasize the independent self and that have high levels of formal schooling. It is plausible that many of the conclusions drawn from research in the Minority World do not apply to the Majority World [104], where interdependence is common, and a full course of formal schooling is not yet the norm.

The prevalence of these cultural preferences—and thus the extent to which assumptions of EF tasks fail to hold across contexts—is a matter for empirical research. It is possible that with the spread of urbanization, schooling, and commerce [88,94,96], and with the forces of globalization [100], the assumptions of EF testing protocols may be shared by increasing numbers of people. It is important, also, to consider the goal of improving ecological validity. If EF tasks are being used as a proxy for performance in formal schools, some assumptions—for example, related to contextualized thinking, abstract representations of stimuli, and dyadic interactions—may be less problematic, because these assumptions are consistent with the norms of classroom behavior.

It is important to note that, aside from issues of ecological validity, EF may develop differently in schooled vs. unschooled populations and in independent vs. interdependent cultures. Schooling and culture may constitute some of the environmental factors contributing to individual differences in EF [105]. We have not discussed this possibility in the paper. Instead, we have focused on the way that biases may be introduced using the same procedures to administer EF tasks in populations with different cultures or different levels of schooling. However, it is possible that observed differences in EF task performance between such populations may be in part due to bias resulting from a mismatch between cultural preferences and task assumptions, and in part due to differences in endogenous EF capacity. Although the literature on the impact of schooling on EF is inconclusive, there is strong evidence that schooling improves general intelligence [106]. It may be challenging to disentangle effects on endogenous EF capacity from bias related to cultural preference, but improving the ecological validity of assessments is a prerequisite.

The issues of ecological validity outlined in this paper can be addressed through researchers taking a systematic approach to considering context when they are adapting and conducting EF assessments. We recommend that to determine the cultural preferences held by a given population, researchers review locally conducted research in anthropology and cultural psychology, supplemented by primary qualitative studies, to investigate the values and preferences of a community (for example, see Jukes et al. [92]). This research can be guided by the principles outlined in this paper—how prevalent schooling is in the community and whether individuals view themselves as independent or interdependent. Based on this research, cultural preferences that may lead to issues of ecological validity can be identified.

We have suggested some adaptations to testing protocols that could help address these issues, including using naturalistic—rather than artificial, decontextualized—tasks; undertaking group assessments, rather than one-on-one assessments; including instructions and examples to make children more comfortable being silly and saying the opposite; focusing on accuracy instead of reaction time; avoiding response-inhibition tasks based on prepotent response assumed to be the norm in the community; and using stimuli with which children are familiar. The success of such adaptations in improving ecological validity should be systematically tested through several steps. Cognitive interviewing [107] can be used to determine whether children understand instructions. Small-sample pilot testing can help address fundamental issues with assessments, such as floor or ceiling effects and children failing to understand, or refusing to comply with, instructions. Larger-scale assessments can be used to determine psychometric properties of assessments, including their validity in predicting performance on everyday tasks, such as academic achievement, and their relation to other assessments, such as adult reports or observation protocols [9]. It would be valuable to test the validity of adapted tests in various populations that differ on factors of theoretical importance—for example, in urban vs. rural populations (as a proxy for independent vs. interdependent cultures) or schooled vs. unschooled populations. Engagement of the participant community in the process of adapting assessments is essential to ensure ecological validity. Community members can help review tasks for face validity [108] and can help ensure that children and assessors share basic assumptions about the testing protocol. Additionally, engaging members of the host community as assessors can help increase alignment between assessor and child in the assumptions of the testing protocol. It is also essential to give voice and agency to the participant community [109] in the design and use of EF assessments, such that assessment adaptations are not imposed from the outside and that community members are genuine partners in the adaptation process. In this process, it can be challenging for community members to recognize what the cultural assumptions of test designers are and where these differ with their own cultural preferences. The hypotheses presented in this paper could be used as a framework to guide conversations between external researchers and the local community to identify problematic assumptions in the testing protocols.

We encourage investigators to do the important work of adapting assessments to new populations. There is an imperative to expand our scientific understanding of EF from the narrow slice of the human population that has participated in EF research to date and to spread the potential benefits of EF assessment and interventions globally. Improving the ecological validity of EF assessments is the first step toward achieving this goal.

## Figures and Tables

**Table 1:** Description of key EF skills, assessment paradigms, and task variants ^1^.

Skill	Paradigm	Task Variants
Inhibitory control–response inhibition	Go/No-Go	Square and Circle; Fish and Shark; Cat and Dog; Animals; different colored squares; Pac-Man/Birds and Ghost/Pork; Cat and Tiger; Grow Your Garden; Emotional
Inhibitory control–response inhibition	Simon Effect (Hearts and Flowers)	Hearts and Flowers; Strawberries and Watermelons; Butterfly and Frog; Spatial Conflict Arrows; Simon Task
Inhibitory control–response inhibition	Hand motoric response	Pencil tap; knock tap; peg tap
Inhibitory control–interference suppression	Stroop	Numerical; Big–Little; Fruit; Silly Sounds; Day and Night
Inhibitory control–interference suppression	Delay of Gratification	Marshmallow Task
Working memory	Corsi/Dot matrix	Memory Game, Mr. Ant, Mr. Peanut, Corsi Blocks, Knox Cube, Geometric Shapes Task, Spatial Delayed Match to Sample Task
Working memory	Digit Span/Word Span	Backward and Forward, Sentence Completion Task, Sentence Repetition
Working memory	Self-Ordered Pointing Task	Self-Ordered Pointing Task
Cognitive flexibility	Flexible Item Selection Task	Something the Same; Triads
Inhibitory control–response inhibition	Head Toes Knees Shoulders	Bear and Dragon
Inhibitory control–response inhibition	Peg Tapping	Knock-Tap, Pencil Tap, Hand Game
Cognitive flexibility	Dimensional Change Card Sort	

^1^ Tasks selected that are appropriate for used globally, based on a selective review of the literature by the Global Executive Function Initiative (GEFI) [19].
